# Analysis of the dihydrofolate reductase-thymidylate synthase gene sequences in *Plasmodium vivax *field isolates that failed chloroquine treatment

**DOI:** 10.1186/1475-2875-9-331

**Published:** 2010-11-18

**Authors:** Won-Ja Lee, Hyung-Hwan Kim, Yien-Kyoung Choi, Kyung-Mi Choi, Mi-A Kim, Jung-Yeon Kim, Jetsumon Sattabongkot, Youngjoo Sohn, Hyuck Kim, Jong-Koo Lee, Han-Sook Park, Hyeong-Woo Lee

**Affiliations:** 1 Division of Malaria and Parasitic diseases, National Institute of Health, Korea Centers for Disease Control and Prevention, Seoul 122-701, Republic of Korea; 2 Vascular Medicine Research Unit, Brigham and Women's Hospital, Harvard Medical School, Cambridge, MA 02139, USA; 3 Department of Entomology, Armed Forces Research Institute of Medical Sciences, Bangkok 10400, Thailand; 4 Department of Gynecology, College of Oriental Medicine, Sangji University, Wonju 220-717, Republic of Korea; 5 International Research Center for Bioscience and Biotechnology, Goesan 367-805, Republic of Korea; 6 Korea Centers for Disease Control and Prevention, Ministry of Health & Welfare, Seoul 122-701, Republic of Korea; 7 Department of Pathology, University of Florida, J-566, 1600 SW Archer Road, Gainesville, FL 32610, USA

## Abstract

**Background:**

To use pyrimethamine as an alternative anti-malarial drug for chloroquine-resistant malaria parasites, it was necessary to determine the enzyme's genetic variation in dihydrofolate reductase-thymidylate syntase (DHFR-TS) among Korean strains.

**Methods:**

Genetic variation of *dhfr-ts *genes of *Plasmodium vivax *clinical isolates from patients who did not respond to drug treatment (*n *= 11) in Korea were analysed. The genes were amplified using the polymerase chain reaction (PCR) with genomic DNA as a template.

**Results:**

Sequence analysis showed that the open reading frame (ORF) of 1,857 nucleotides encoded a deduced protein of 618 amino acids (aa). Alignment with the DHFR-TS genes of other malaria parasites showed that a 231-residue DHFR domain and a 286-residue TS domain were seperated by a 101-aa linker region. This ORF shows 98.7% homology with the *P. vivax *Sal I strain (XM001615032) in the DHFR domain, 100% in the linker region and 99% in the TS domain. Comparison of the DHFR sequences from pyrimethamine-sensitive and pyrimethamine-resistant *P. vivax *isolates revealed that nine isolates belonged to the sensitive strain, whereas two isolates met the criteria for resistance. In these two isolates, the amino acid at position 117 is changed from serine to asparagine (S117N). Additionally, all Korean isolates showed a deletion mutant of THGGDN in short tandem repetitive sequences between 88 and 106 amino acid.

**Conclusions:**

These results suggest that sequence variations in the DHFR-TS represent the prevalence of antifolate-resistant *P. vivax *in Korea. Two of 11 isolates have the Ser to Asn mutation in codon 117, which is the major determinant of pyrimethamine resistance in *P. vivax*. Therefore, the introduction of pyrimethamine for the treatment of chloroquine-resistant vivax malaria as alternative drug in Korea should be seriously considered.

## Background

*Plasmodium vivax*, a causative agent of relapsing benign tertian human malaria, is the second- most important human malaria and afflicts several hundred million people annually [1]. The disease is a major public health problem with associated socioeconomic ramifications for many temperate and most tropical countries.

Vivax malaria has been prevalent throughout the Korean peninsula for several centuries. However, as a result of a national malaria eradication program, and with help from the World Health Organization (WHO), the incidence of vivax malaria has rapidly decreased [2,3]. Vivax malaria was believed to have been eradicated in the Republic of Korea (ROK) since the late 1970 s, although two sporadic cases were detected in the 1980 s [4]. These two patients relapsed after a long incubation. In 1993, a vivax malaria case was diagnosed among ROK army soldiers serving in the northern Gyeonggi-do [5]. Subsequently, Cho *et al *reported two civilian patients infected with vivax malaria [6]. Thereafter, a total of 2,198 patients (1,548 soldiers and 650 civilians) were detected from 1994 to 1997 near the demilitarized zone (DMZ), centering around Paju-shi, Yonchon-gun, Chorwon-gun, Kimpo-shi, Kangwha-gun, Koyang-shi, and Dongducheon-shi. Hence, the possibility of the re-establishment and geographical expansion of malaria is highly concerning [7].

Chloroquine has been the standard treatment for blood-stage vivax malaria for over more than 40 years, whereas primaquine has been exploited for preventing relapses of the liver-stage hypnozoites. However, chloroquine-resistant *P. vivax *has been reported in several parts of the world [8-13], and relapse parasitaemia due to primaquine-resistant liver-stage hypnozoites has become increasingly widespread [14]. To manage chloroquine-resistant malaria, antifolate drugs including pyrimethamine, proguanil, and cycloguanil, in monotherapy and later in combination with sulphonamides, have been frequently employed as alternative anti-malarial agents in endemic areas. However, a few years after the introduction of antifolates to combat chloroquine resistance, clinical observations of slow parasite clearance and early reappearance of parasitemia after the therapy were reported, especially in *P. vivax *infections [15,16].

Pyrimethamine is an analogue of dihydrofolate that inhibits dihydrofolate reductase (DHFR; 5,6,7,8-tetrahydrofolate: NADP+ oxidoreductase, EC 1.5.1.3). As in other protozoa, malarial DHFR is physically linked to thymidylate synthase (TS; EC 2.1.45), making DHFR-TS a bifunctional enzyme. Malaria parasites are dependent on this enzyme for folate biosynthesis. Specific inhibition of the DHFR domain of the enzyme by pyrimethamine blocks pyrimidine biosynthesis, leading to inhibition of DNA replication [17]. Resistance to antifolate agents, possibly due to mutations at specific sites in DHFR, has been well studied in *P. falciparum *[18-21]. The loss of susceptibility to these antifolates results from mutations associated with reduced drug binding affinity to the active site of the DHFR enzyme [22]. A number of mutant forms of *P. vivax *dihydrofolate reductase (*dhfr*) have also been reported in various geographical regions [23-25]. These mutant *dhfr *of *P. vivax *encode amino acid sequences similar to those involved in antifolate resistance in *P. falciparum *and are responsible for antifolate resistance in a fashion similar to that previously reported in *P. falciparum *malaria [26,27].

Analysis of *dhfr *mutations in wild isolates has been considered as a valuable molecular approach for resistance mapping and the monitoring of malaria control measures [28,29]. In fact, the regimen of chloroquine (2,000 mg/3 days) and primaquine (15 mg/day for 14 days) has been used to treat vivax malaria patients since the re-emergence of *P. vivax *in Korea. However, the number of cases failing to respond to drug treatment has increased annually, and chloroquine-resistant malaria parasites have begun to spread nationally (Korea Centers for Disease Control and Prevention). Therefore, it is urgent that the *dhfr-ts *sequences be investigated before introducing antifolate drugs to control chloroquine-resistant malaria in Korea. Here, we first report the full gene sequences of DHFR-TS of *P. vivax *Korean isolates. These validations of molecular epidemiological efforts to study *P. vivax *in different endemic areas may be useful to establish an epidemiological map of drug-resistant vivax malaria and to update anti-malarial policy guidelines in Korea.

## Methods

### Blood collection

Blood samples were collected from patients who experienced a relapse or re-infection. Thin and thick blood smears were prepared from patients' fingertips for microscopic diagnosis, after which additional blood samples were collected from the patients confirmed to be infected for further study. Informed consent was obtained from all patients. All samples were collected under human-use protocols that have been reviewed and approved by the Human Ethics Committee of the National Institute of Health in Korea.

### Extraction of parasite DNA

*Plasmodium vivax *genomic DNA was extracted from the patients' blood using a QIAamp DNA Blood Kit (Qiagen, Valencia, USA) following the manufacturer's instructions. To confirm the diagnosis and to differentiate the infections caused by *P. vivax*, a single-step polymerase chain reaction (PCR) was performed as described previously [30].

### Cloning and sequence analyses of the *dhfr-ts *gene

Polymerase chain reaction was done with *P. vivax *genomic DNA as a template, with specific primers for *dhfr-ts *to obtain the region between the start codon (ATG) and the stop codon (TAA): DHFR-F1 (30 mer), 5'-ATGGAGGACCTTTCAGATGTATTTGACATT-3'; and DHFR-R (30 mer), 5'-TTAGGCGGCCATCTCCATGGTTATTTTATC-3'. The amplification reaction was set up in a total volume of 50 ul, containing 100 ng genomic template DNA, 2 mg MgSO_4_, 200 μM each dNTP and 1.5 U of *Taq *polymerase. The PCR was performed for 34 cycles: the first cycle consisted of 94°C for 5 min; the subsequent 34 cycles consisted of 94°C for 1 min, 58 °C for 2 min, 72°C for 2 min; and the final cycle consisted of 72°C for 10 min. Amplified products were gel-purified, ligated into the pCR2.1 vector and transformed into competent *Escherichia coli *Top10 cells, using a TOPO TA Cloning Kit (Invitrogen, Carlsbad, USA). Sequencing reactions were done using the BigDye Terminator Cycle Sequencing Ready Reaction Kit in an ABI 377 automatic DNA sequencer (Applied Biosystems, Foster City, USA). To determine the full gene sequences of *dhfr-ts*, two internal forward primers were designed: DHFR-F2 (30 mer), 5'-CTGAAGTACTACAAATGCTTCATCATTGGG-3', and DHFR-F3 (30 mer), 5'-AGGCAGAGGAAGACGACCTCGTGTACTTCA-3'. For verification of the sequences, multiple plasmid clones containing each insert were analysed. Nucleotide and deduced amino acid sequences were analysed with the SeqEd.V1.0.3 program and the CLUSTAL V method of the Megalign programme, a multiple-alignment programme of the DNASTAR package (DNASTAR, Madison, USA).

## Results

### Failed cases of chloroquine-primaquine combined treatment in Korea from 1996 to 2001

After a two-decades-long absence of vivax malaria in Korea, and starting with one patient who had served in the demilitarized zone (DMZ) as a soldier in 1993, the incidence of malaria increased rapidly [3-7]. To treat the vivax malaria patients, a combined chloroquine (2000 mg/3 days) and primaquine (15 mg/day for 14 days) regimen has been used as the standard treatment. Three years after the introduction of this standard regimen, in 1996, two cases failed to respond to the combination. Thereafter, 304 of 9,918 patents (3.07%) failed to be cured using this standard regimen (Table 1, Centers for Disease Control and Prevention, Korea).

**Table 1:** Failed cases of anti-malaria drug treatment

	1996	1997	1998	1999	2000	2001	Total
Number of patients	71	568	2275	2537	2853	1614	9918

Failed treatment cases	2	12	45	65	121	59	304

Rate (%)	2.81	2.11	1.98	2.56	4.24	3.67	3.07

### Cloning of the *dhfr -ts *genes of *P. vivax *Korea isolates

A total of 11 blood samples were collected from relapsed patients who visited the Public Health Care Centers located in Gyeonggido and Gangwondo in the malaria epidemic area of Korea. Microscopic examination followed by single-step PCR analysis revealed that all relapsed patients were infected with *P. vivax*. Several primers were synthesized - based on conserved regions in the DHFR and TS domains of the malarial DHFR-TS gene - to generate overlapping fragments of *P. vivax *genomic DNA. Full DHFR-TS gene PCR amplification products were obtained from *P. vivax *genomic DNA using the synthetic oligonucleotide primer pairs DHFR-F1 and DHFR-R. All of the PCR products were 1,857 bp. The complete DNA sequence of the DHFR-TS genes of *P. vivax *isolates were obtained by assembling the overlapping sequences produced using the DHFR-F1, DHFR-F2, DHFR-F3, and DHFR-R primers as described in Methods. To demonstrate that the four fragments were from the same gene, DHFR-F1 and DHFR-R primers hybridizing to the 5' and 3' ends, respectively, were synthesized to amplify, clone, and sequence the entire DHFR-TS gene. These primers included the methionine initiation codon and the stop codon. The combined nucleotide sequences from the PCR fragments contained a single open reading frame (ORF) of 1,857 bp encoding a deduced gene product of 618 aa. The nucleotide sequences encoding each gene were submitted to GenBank (Accession numbers; DQ514918, DQ514919, DQ514920, DQ514921, DQ517894, DQ517895, DQ517896, DQ517897, DQ517898, DQ517899 and DQ517900).

### Comparison of the deduced DHFR-TS amino acid sequence of Korean *P. vivax *isolates with other species

**Figure 1:**
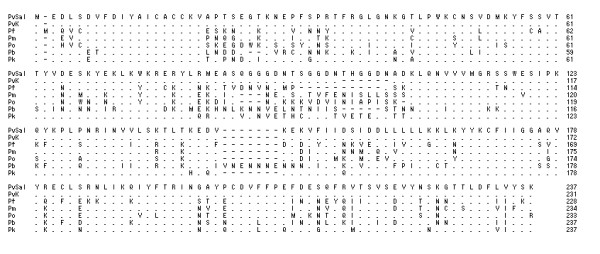
**Comparison of the DHFR domains of several species**. PvSal; *P. vivax *Sal I strain (GenBank accession no. XM001615032), PvK; *P. vivax *Korean isolate 1063 (DQ517897), considered to represent the wild-type sequence, Pf; *P. falcipaum *(J03028), Pm; *P. malariae *(AY846633), Po; *P. ovale *(EU266606), Pb; *P. berghei *(XM673777), Pk; *P. knowlesi *(XM002258192).

**Figure 2:**
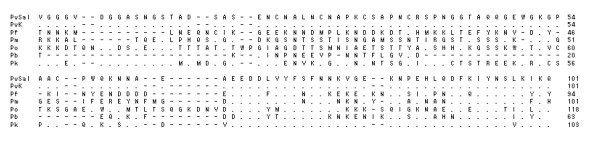
**Comparison of the linker regions of several species**. PvSal; *P. vivax *Sal I strain (GenBank accession no. XM001615032), PvK; *P. vivax *Korean isolate 1063 (DQ517897), considered to represent the wild-type sequence, Pf; *P. falcipaum *(J03028), Pm; *P. malariae *(AY846633), Po; *P. ovale *(EU266606), Pb; *P. berghei *(XM673777), Pk; *P. knowlesi *(XM002258192).

**Figure 3:**
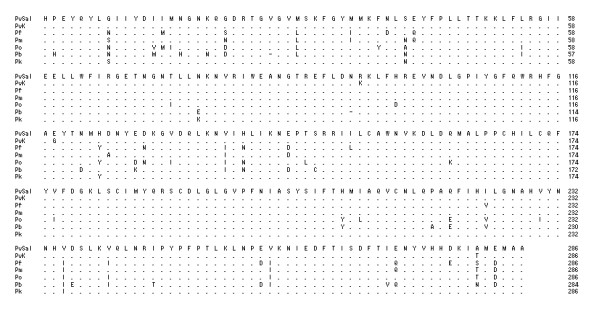
**Comparison the TS domain from several species**. PvSal; *P. vivax *Sal I strain (GenBank accession no. XM001615032), PvK; *P. vivax *Korean isolate 1063 (DQ517897), considered to represent the wild-type sequence, Pf; *P. falcipaum *(J03028), Pm; *P. malariae *(AY846633), Po; *P. ovale *(EU266606), Pb; *P. berghei *(XM673777), Pk; *P. knowlesi *(XM002258192).

According to amino acid homology, the Korean *P. vivax *isolates contained a single gene of interest with three structural domains: a 5' end DHFR domain (bp 1-693), a linker region (bp 694-996; 302 total bp), and a 3' end TS domain (bp 997-1,857; 860 total bp). In all protozoan parasites examined so far, DHFR and TS make up a bifunctional enzyme encoded by a single gene. The Korean *P. vivax *isolate is thus not an exception to this general rule. In the DHFR domain of Korean isolate (Genbank accession no. DQ517897), 98.7%, 64.5%, 70.6%, 73.4%, 54.5% and 56.5% of the residues were identical between the enzymes of *P. vivax *Sal I strain (XM001615032, 1,880 bp), *Plasmodium falciparum *(J03028, 2,160 bp), *Plasmodium malariae *(AY846633, 1,866 bp), *Plasmodium ovale *(EU266606, 1,914 bp), *Plasmodium berghei *(XM673777, 1,758 bp), and *Plasmodium knowlesi *(XM002258192, 1,881 bp), respectively (Figure 1). In the linker region of Korean isolate (DQ517897), 100%, 23.4%, 26.6%, 29.7%, 28.6%, and 31.7% of the residues were identical between the enzymes of *P. vivax *Sal I strain, *P. falciparum*, *P. malariae*, *P. ovale*, *P. berghei*, and *P. knowlesi*, respectively (Figure 2). In the TS domain of Korean isolate (DQ517897), 99%, 92.3%, 94.8%, 89.9%, 87.3%, and 87.7% of the residues were identical between the enzymes of *P. vivax *Sal I strain, *P. falciparum*, *P. malariae*, *P. ovale*, *P. berghei*, and *P. knowlesi*, respectively (Figure 3).

### Comparison of the deduced DHFR-TS amino acid sequence of Korean *P. vivax *isolates with other *P. vivax *isolates

When comparing the deduced amino acid sequences of DHFR-TS between *P. vivax *Sal I (XM001615032) and all Korean isolates, this gene shows high homology, from 97.8-99.6% in the DHFR domain, 98-100% in the linker region, and 97.8-99.6% in the TS domain. Additionally, comparison of the deduced amino acid sequences indicated a high level of conservation in several regions between different isolates: Pakistan (X98123), Surinam (AJ003074), Thailand (AJ003051), Cambodia (AJ003071), Comoros islands (AJ003073), Indonesia (AJ003077), and Madagascar (AJ003076). In the DHFR domain of Korean isolate (DQ517897), 97.0%, 95.4%, 95%, 95%, 94.6%, 94.6%, and 86.4% of the residues were identical between the enzymes of Cambodia, Surinam, Madagascar, Comoros islands, Thailand, Indonesia, and Pakistan, respectively [23].

**Figure 4:**
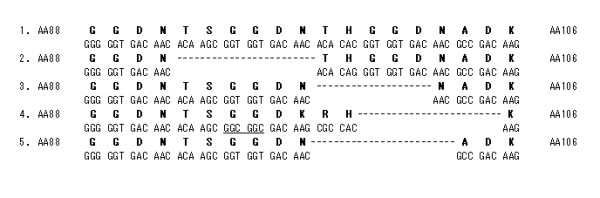
**Polymorphism of the short tandem repetitive sequences (GGDN) within the DHFR domain of the *P. vivax *DHFS-TS gene**. (1) corresponds to the consensus sequence (amino acid residue 88-106) found in 15 of 30 isolates; (2) is the deleted sequence from isolates PHI/Djibouti and LGF/India. The latter isolates had a silent mutation at the nucleotide level (CAC→CAG); (3) represents the deleted sequence of isolate VAN/Thailand and 11 Burmese isolates; (4) corresponds to the deleted sequence from isolate LFT/Cambodia. Two identical silent mutations (GGT→GGC) and two mutations in 97 (Asn→Lys) and 98 (Thr→Arg) were detected [23]; (5) corresponds to the deleted sequence from Korean isolates (n = 11). Deletions are denoted by dashes.

The *P. vivax dhfr *gene contains a unique tandem repeat region between amino acids 88 and 106. Four allelic variants that differed with respect to the repeat motifs were previously identified [23]. The sequence THGGDN (18 bp) was missing in all Korean isolates (Figure 4).

### Comparison of the sequences from pyrimethamine-sensitive and pymethamine-resistant parasites

The 12 non-synonymous point mutations in the *dhfr *domain of *P. vivax *have been reported [23,24,29]. Among them, mutations at codons 57 (F to L/I), 58 (S to R), and 117 (S to N/T) encode amino acids similar to those which cause antifolate resistance in *P. falciparum*, suggesting that antifolate resistance in *P. vivax *might have arisen in the same manner as that recognized in *P. falciparum *[24,27]. Nine of the studied Korean isolates had the wild-type genotype, whereas two isolates (KNIH98-55 and KNIH99-3170) had the single mutation S117N, which is key mutation associated with pyrimethamine resistance [17,27]. However, these resistant strains have amino acids similar to those in sensitive strains at codons 33 (P), 57 (F), 58 (S), 61 (T), and 173 (I). The nine wild-type isolates have exactly the same amino acids as the sensitive strain.

## Discussion

Only three years after the re-emergence of vivax malaria in Korea in 1993, it was revealed that the combined standard regimen of chloroquine and primaquine was not effective in all cases. Therefore, it is necessary to introduce another drug to control the chloroquine-resistant strains. One class of candidates is antifolate drugs, but strains resistant to this treatment have spread worldwide. To find a reliable treatment, it should be investigated the current status of antifolate drug resistance in vivax malaria.

Korean isolate (KNIH99-1063, DQ517897) shows 98.7% homology rate with *P. vivax *Sal I strain (XM001615032) in the DHFR domain, 100% in the linker region and 99% in the TS domain (Figure 1). The TS domain shows high homology among different species (Pf, Pm, Po, Pb, and Pk; 87.3%-94.8%) (Figure 3), however low homology rates are observed in the linker region (23.4% to 31.7%) (Figure 2). The DHFR domains show an intermediate homology rate, from 54.5% to 73.4% (Figure 1).

The relatively rapid propagation of antifolate-resistant *P. vivax *might be explained by this species' intrinsic resistance to antifolate drugs such as pyrimethamine. However, resistance acquired through sequential appearance of point mutations due to drug pressure and to the progressive selection of resistant parasites is a more plausible explanation for the failure of treatment of vivax malaria [31]. Molecular studies associated with clinical observations of vivax malaria have demonstrated that the major mechanism of antifolate resistance results from specific mutations in the *dhfr *gene of the parasite [24,29]. Although a total of 12 nonsynonymous mutations were found in *P. vivax dhfr*, based on secondary-structure analysis and homology modeling to *P. falciparum dhfr*, biochemical analysis of recombinant enzymes and clinical correlation demonstrated four point mutations at amino acid residues 57, 58, 61, and 117. These are thought to be critically involved in pyrimethamine resistance [24,27,29].

Two of 11 Korean isolates have the S117N single mutation. This single S117N mutation in PvDHFR conferred about 4000- and 1600-fold increased resistance to pyrimethamine and cycloguanil, respectively, compared to the wild-type PvDHFR [27]. The S58R/S117N double mutant PvDHFR was 10-to 25-fold less resistant than the S117N mutant to the inhibitors. Antifolate had never been used for the control of re-emerging vivax malaria in Korea, so we doubted about it. Interestingly, this single mutated allele (S117N) was identified at a high frequency in isolates in samples from Turkey and Azerbaijan (36% and 71% of isolates, respectively), areas where antifolate drug pressure of resistance is not obvious; this allele was not found in Indonesia (n = 36) or Thailand (n = 16). Additionally, it appears that *P. vivax *has developed resistance to sulfadoxine-pyrimethamine more rapidly than *P. falciparum *has; hence, artesunate plus sulfadoxine-pyrimethamine may not be effective overall against *P. vivax *in many areas [32].

The first-line treatment against *P. vivax *malaria in these areas is still the combination of chloroquine-primaquine, like in Korea, and a recent clinical study has demonstrated that treatment with chloroquine followed by treatment with primaquine is 100% effective in patients from Azerbaijan [33]. Therefore, it needs intensive molecular epidemiological studies in Korea with lots of samples. The easiest and most effective way might be to apply the restriction fragment length polymorphism (RFLP) analysis of the *P. vivax *DHFR gene as introduced by Imwong *et al *[24].

If the spread of chloroquine resistance encourages health care policy administators to move toward antifolate drug regimens as first-line treatment, data such as ours offer a snapshot of the prevalence of DHFR mutations in multiple geographical areas and can provide crucial information on the potential appearance of sulfadoxine-pyrimethamine resistance in Korea.

## Conclusions

Together with these data, antifolate-resistant *P. vivax *is already starting to spread in Korea. Further detailed geographical mapping of current and changing patterns of drug resistance in vivax malaria on a national or regional scale would prove a valuable aid to developing and updating national anti-malarial policy guidelines in Korea. Control measures and inter-governmental co-operation are also needed to block the spread of drug-resistant malaria in the country.

## Competing interests

The authors declare that they have no competing interests.

## Authors' contributions

HWL, HHK, and WJL conceived and designed the study and contributed to the execution of the research. HWL wrote the manuscript. YKC, JYK, HK, and YS collected the blood samples in the field. YKC, MAK, HSP, and KMC performed DHFR-TS gene cloning and sequencing. JS, HWL, JKL, and WJL helped in the design of the study and genetic analysis for the DHFR-TS gene. All authors have read and approved the final manuscript.
